# Predictive potential of Nomogram based on GMWG for patients with hepatocellular carcinoma after radical resection

**DOI:** 10.1186/s12885-021-08565-2

**Published:** 2021-07-15

**Authors:** Liying Ren, Dongbo Chen, Wentao Xu, Tingfeng Xu, Rongyu Wei, Liya Suo, Yingze Huang, Hongsong Chen, Weijia Liao

**Affiliations:** 1 Laboratory of Hepatobiliary and Pancreatic Surgery, Affiliated Hospital of Guilin Medical University, Guilin, 541001 Guangxi People’s Republic of China; 2 Peking University People’s Hospital, Peking University Hepatology Institute, Beijing Key Laboratory of Hepatitis C and Immunotherapy for Liver Disease, 100044 Beijing, People’s Republic of China

**Keywords:** Hepatocellular carcinoma, Radical resection, Prognosis, Nomogram

## Abstract

**Background:**

Since it’s a challenging task to precisely predict the prognosis of patients with hepatocellular carcinoma (HCC). We developed a nomogram based on a novel indicator GMWG [(Geometric Mean of gamma-glutamyltranspeptidase (GGT) and white blood cell (WBC)] and explored its potential in the prognosis for HCC patients.

**Methods:**

The patients enrolled in this study were randomly assigned to training and validation cohorts. And we performed the Least Absolute Shrinkage and Selection Operator proportional hazards model (LASSO Cox) model with clinical characteristics, serum indexes, and novel GMWG. Multivariate analysis was performed to build a nomogram. The performance of the nomogram was evaluated by C-index, the area under the receiver operating characteristic curve (AUC), and the calibration curve. Kaplan-Meier curves showed discrimination of the nomogram. Clinical utility was assessed by decision curve analysis (DCA). The discrimination ability of the nomogram was determined by the net reclassification index (NRI).

**Results:**

The geometric mean of GGT and white WBC count (GMWG), neutrophil to lymphocyte ratio (NLR), and tumor size were significantly associated with the overall survival (OS). The variables above were used to develop the nomogram. The indexes of nomogram were 0.70 and 071 in the training or validation cohort, respectively. AUC of 1-, 3- and 5-year OS showed satisfactory accuracy as well. The calibration curve showed agreement between the ideal and predicted values. Kaplan-Meier curves based on the overall survival (OS) and disease-free survival (DFS) showed significant differences between nomogram predictive low and high groups. DCA showed clinical utilities while NRI showed discrimination ability in both training or validation cohort.

**Conclusions:**

GMWG might be a potential prognostic indicator for patients with HCC. The nomogram containing GMWG also showed satisfaction prediction capacity.

**Supplementary Information:**

The online version contains supplementary material available at 10.1186/s12885-021-08565-2.

## Background

Hepatocellular carcinoma (HCC) is the sixth common cancer and the fourth leading cause of cancer-related death and has been a growing public health issue [1]. There are many risk factors that might lead to HCC, such as alcohol, viral hepatitis, and hepatic cirrhosis [2]. Radical resection is considered as an effective strategy which may curatively improve the prognosis of patients with early-stage HCC [3–5]. However, due to the insidious onset of HCC, many patients had lost the chance to get surgery or liver transplantation before they were diagnosed. There are strict requirements for patients in liver transplantation and can hardly be widely used. Despite the fact that transarterial chemoembolization (TACE) and radiofrequency ablation bring other options for HCC patients, the clinical outcome is still not promising.

With the use of immune checkpoint inhibitors (ICIs) in other tumors [6], people hoped to achieve similar benefits in HCC to improve the prognosis of patients, but the clinical response rate is only about 15–20% [7, 8]. In clinical practice, it’s a challenging task to precisely predict the prognosis of patients with HCC. Although there is some progress in the prediction of postoperative outcomes, the prognosis of patients with HCC remains poor. Therefore, there is an urgent need for convenient and readable indicators or tools to predict the prognosis of postoperative patients. Nomogram is a readable visual tool, which is mainly used to diagnose or predict the prognosis of patients by summarizing the results of several daily clinical examination results. Nomogram is widely developed in different types of cancers among patients with different conditions [9, 10], which is also applicable in HCC. Therefore, how to construct and screen out more valuable indicators is the point of clinical researchers’ work. However, for each variable in the conventional nomogram, there will be an optimal cutoff value determined, which might differ from the different cohorts or many other factors. In order to ensure the repeatability of the nomogram, each parameter is analyzed as a numerical variable in this study, and the distribution level can be clearly observed.

Serological indicators play a crucial role in the diagnosis or prognosis prediction in patients with HCC [11]. Many indicators have been explored or developed to predict the prognosis of patients with HCC. Moreover, we have verified several indexes in our previous work [12–14]. Since primary liver cancer is considered to be an immunosuppressive tumor so that the indicators that reflect immune response or liver function have always been focused on by researchers [15]. Integrated indicators such as neutrophil to lymphocyte ratio (NLR) and systemic immune-inflammation index (SII) [16, 17], both serve as biomarkers of baseline inflammatory response and have been developed to predict the outcome of HCC patients. However, few indicators could simultaneously evaluate the immune status and liver function. The increase of white blood cell count might indicate an inflammatory response to infection or tumor. At the same time, GGT is a membrane-binding enzyme which is considered a signal of normal liver cell damage.

Herein, we construct a novel indicator, the Geometric Mean of gamma-glutamyltranspeptidase (GGT) and white blood cell (WBC) (GMWG). Next, a nomogram was developed base on GMWG to predict prognosis for patients with HCC who underwent surgery and also showed satisfaction prediction capacity comparing with other models or stage systems. Our findings offer new options for predicting the outcome of patients with HCC.

## Materials and methods

### Patients

The patients were divided into training cohort and validation cohort by setting seed in R. Demographic characteristics, clinicopathological data, laboratory examination were collected, and the contents included personal history (gender, age, smoking and drinking history, etc.), pathologic features (tumor size, count, vascular invasion lymph node metastasis, hepatitis, cirrhosis and the degree of cell differentiation, etc.) and the latest hematological examinations before operation (hepatitis B surface antigen (HBsAg), alpha fetoprotein (AFP), liver function, blood routine, blood biochemistry, etc.).

### Diagnostic criteria and exclusion criteria

All patients underwent auxiliary examinations such as imaging examination and hematological examinations before the operation, and postoperative pathologically examine was diagnosed as HCC. Imaging examination including at least one of ultrasonography (US), computerized tomography (CT), magnetic resonance imaging (MRI). Patients were categorized according to the American Joint Committee on Cancer (AJCC) TNM staging system. Curative resection was defined as complete removal of the tumor, no residual tumor or new lesion observed in two observations at an interval of no less than 4 weeks. All tumor specimens were histopathologically examined by two independent pathologists. The main exclusion criteria of this study were as follows: 1) received other anti-tumor therapies prior to the surgery; 2) history of other cancers; 3) died during the perioperative period; 4) with hematological system diseases or severe infection that might influence examinations results; 5) incomplete clinical data, and lost contact in the follow-up period.

### Follow-up

We conducted regular postoperative follow-up for patients included via outpatient reexamination or telephone. The methods were as follows: Blood routine, liver and kidney function, and abdominal ultrasonography were examined every 2 months within the first 2 years while every 3–6 months after 2 years. CT contrast-enhanced scanning or MRI examination were performed if the reexamination results were abnormal. Overall survival (OS) was determined as the interval between the date of operation and the date of death or the last follow-up date, while disease-free survival (DFS) was determined from the date of radical surgery to the date of the first recurrence at any site, death or the last follow-up date.

### Statistical analysis

Continuous variables which conforming to normal distribution were shown as mean ± standard deviation (SD) and were compared by Student’s *t*-test. Chi-square tests were used to compare categorical variables. Kaplan-Meier method and Log-rank test were conducted to analyze the different survival rates among different groups. The Cox regression analysis was used for multivariate analyses, and the nomogram was built via rms and regplot packages. The receiver operating characteristic (ROC) curves based on the timeROC package were used to define sensitivity, specificity. The Least Absolute Shrinkage and Selection Operator proportional hazards model (LASSO Cox) regression model analysis depended on the glmnet package, while the nomogram and calibration curve were established by the rms package. Decision curve analysis (DCA) was based on the rmda package and net reclassification improvement (NRI) was calculated by nricens package. SPSS18.0 (SPSS Inc., Chicago, IL) and R version 4.0.3 (https://www.rproject.org/) were used for statistical analysis, and *P <* 0.05 was considered statistically significant.

### Definition of integrated indicators

GMWG was defined as the geometric mean of gamma-glutamyltranspeptidase and white blood cell count, $GMWG=\sqrt{GGT\ast WBC}$; GLR was defined as the ratio of gamma-glutamyltranspeptidase and lymphocytes count, *GLR* = *GGT*/*LYMPH*; NLR was defined as the ratio of neutrophils count and lymphocytes count, *NLR* = *NEUT*/*LYMPH*; SII was defined as the systemic immune-inflammation index and was used to compare with the nomogram, *SII* = *PLT* ∗ *NEUT*/*LYMPH*, where PLT, NEUT and LYMPH were platelet, neutrophil and lymphocyte counts, respectively. All the variables above are analyzed as continuous variables.

## Results

### Clinicopathologic characteristics and survival of the patients with HCC

From April 2008 to September 2015, a total of 516 HCC patients who went curative resection in the Affiliated Hospital of Guilin Medical University (Guilin, China) were enrolled in this study according to exclusion criteria with clinicopathological data and serum indexes attached. The laboratory and clinical characteristics of the patients are as follow (Table 1) (Table S1). Most HCC patients were male (87.0 and 85.6%, respectively). Most patients were positive for HBV surface antigen (82.8 and 80.8%) and had cirrhosis (90.8 and 92.5%). Most of the variables showed no significant difference between the training and the validation cohort. Microvascular invasion (MVI) was present in 90 (24.3%) and 29 (19.7%) patients in the training validation cohorts, respectively (Table 1). The mean follow-up times were 62.0 and 63.0 months. Median overall survival times in the two groups were both 55.0 months while median disease-free survival times were 27.0 and 24.0 months. The 5-year survival rates were 44.9 and 47.1%, the recurrence rates were 41.0 and 41.1%, respectively. All the information above indicates that the patients in the training and validation cohort have a balanced survival distribution and baseline clinical characteristics.

**Table 1:** The clinicopathologic characteristics of patients in the training and validation cohorts

Parameter	Training cohort	Validation cohort	***p*** value
	(***n*** = 370)	(***n*** = 146)	
Gender: female/male (n)	48/322	21/125	0.104
Age (years)	51.74 ± 11.77	50.78 ± 10.89	0.261
HBsAg: negative/positive (n)	54/306	28/118	0.248
Family history: absent/present (n)	332 /38	124/22	0.126
Drinking: absent/present (n)	212/158	75/71	0.222
Smoking: absent/present (n)	218/152	88/58	0.778
Cirrhosis: absent/present (n)	34/336	11/135	0.548
MVI: absent/present (n)	280/90	117/29	0.279
NEUT (×10^9^/L)	3.90 ± 1.93	4.10 ± 2.36	0.436
LYMPH (×10^9^/L)	1.69 ± 0.61	1.66 ± 0.62	0.057
Platelets (×10^9^/L)	186.33 ± 86.03	183.56 ± 78.82	< 0.001*
GGT (U/L)	106.97 ± 120.92	104.40 ± 109.25	< 0.001*
LDH (U/L)	215.43 ± 95.46	204.79 ± 90.36	0.113
Tumor size (cm)	7.65 ± 4.54	7.97 ± 4.84	0.140
Tumor number: single/multiple (n)	280/50	108/38	0.239
Grade: G1/G2/G3 (n)	54/211/105	27/100/19	< 0.001*
LNM: absent/present (n)	10/360	7/139	0.230
Child-Pugh stage: A/B (n)	328/42	131/15	0.725
AFP ≤ 20/ > 20 (ng/mL)	130/240	53/93	< 0.001*
NLR	2.63 ± 2.17	2.81 ± 2.01	0.028*
GLR	74.86 ± 99.5	73.51 ± 82.8	0.115
GMWG	23.50 ± 12.32	23.6 ± 12.06	0.301

Abbreviations: *n* number of patients, *HBsAg* hepatitis B surface antigen, *MVI* microvascular invasion, *NEUT* neutrophil count, *LYMPH* lymphocyte count, *GGT* gamma-glutamyl transpeptidase, *LDH* lactate dehydrogenase, *LNM* lymph node metastasis, *AFP* alpha fetoprotein, *NLR* neutrophils to lymphocytes ratio, *GLR* gamma-glutamyl transpeptidase to lymphocyte count ratio, *GMWG* geometric mean of gamma-glutamyl transferase and white blood cell

*p-value indicates statistically significant

### LASSO cox and multivariate analysis of the clinical indicators

**Fig. 1:**
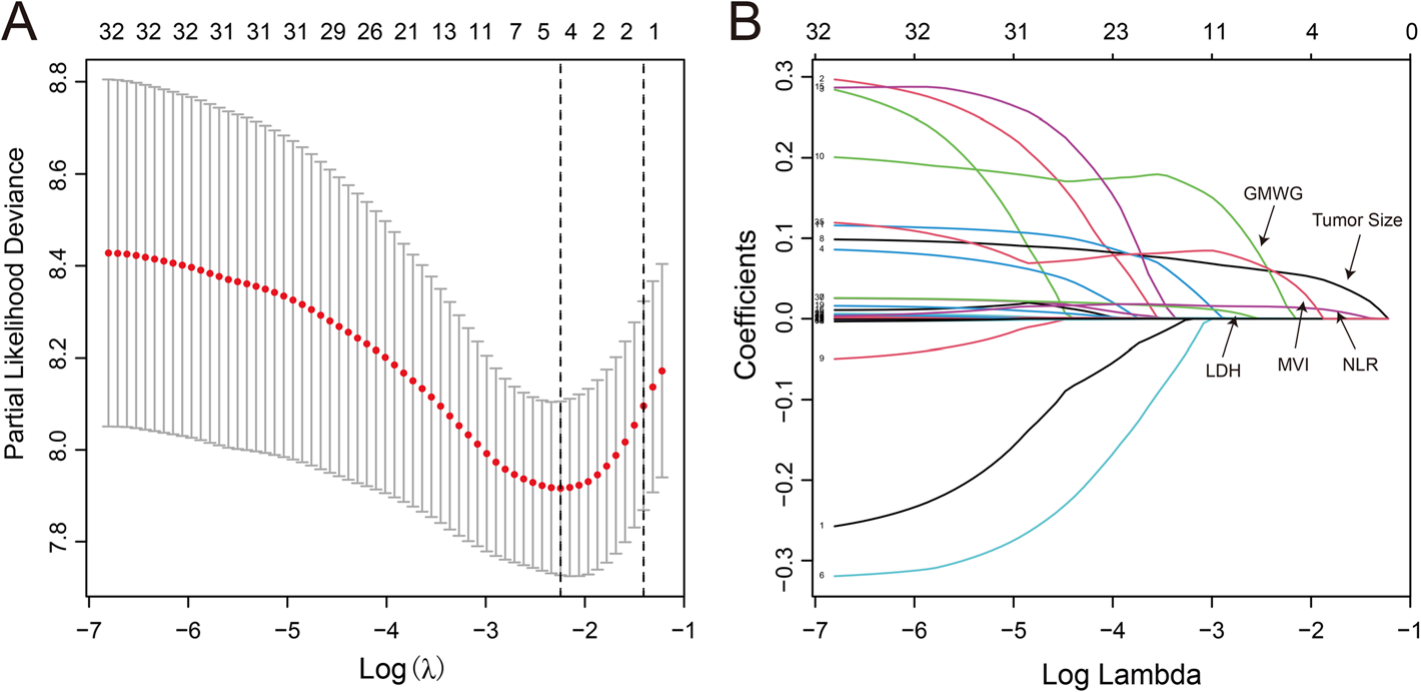
Clinical indicators selection using the LASSO Cox regression model. **A** log (lambda) and partial likelihood deviance were shown, the dotted line is displayed at the minimum log (lambda) represents the optimal number of predictors. **B** LASSO coefficients of total 32 clinical indicators. Nonzero coefficients were determined based on the optimal log (lambda)

A total of 370 patients with clinical variables in training cohort were included in LASSO Cox model to avoid the influence of confounding factors. Five variables left with nonzero coefficients according to the minimum criteria (Fig. 1 A-B). Next, we performed multivariate analysis among the five variables above, and found GMWG (HR = 1.02; 95% CI = 1.01–1.03; *P <* 0.001), NLR (HR = 1.11; 95% CI = 1.05–1.18; *P <* 0.001) and tumor size (HR = 1.08; 95% CI = 1.05–1.11; *P <* 0.001) were significant corelated with overall survival (Table 2).

**Table 2:** Multivariate Cox Regression Analyses of Variables Associated with Overall Survival

Variable	β	Hazard Ratio (95% CI)	***p*** value
Tumor size	0.05670	1.08 (1.05–1.11)	**<** 0.001*
MVI	0.01544	1.30 (0.97–1.76)	0.082
LDH	0.00029	1.01 (1.00–1.01)	0.086
NLR	0.04943	1.11 (1.05–1.18)	**<** 0.001*
GMWG	0.01446	1.02 (1.01–1.03)	**<** 0.001*

Abbreviations: *β* coefficients of multivariate Cox regression, *CI* confidence interval, *MVI* microvascular invasion, *LDH* layered double hydroxide, *NLR* neutrophils to lymphocytes ratio, *GMWG* geometric mean of gamma-glutamyl transferase and white blood cell

*p-value indicates statistically significant

### Development and assessment of predictive nomogram

**Fig. 2:**
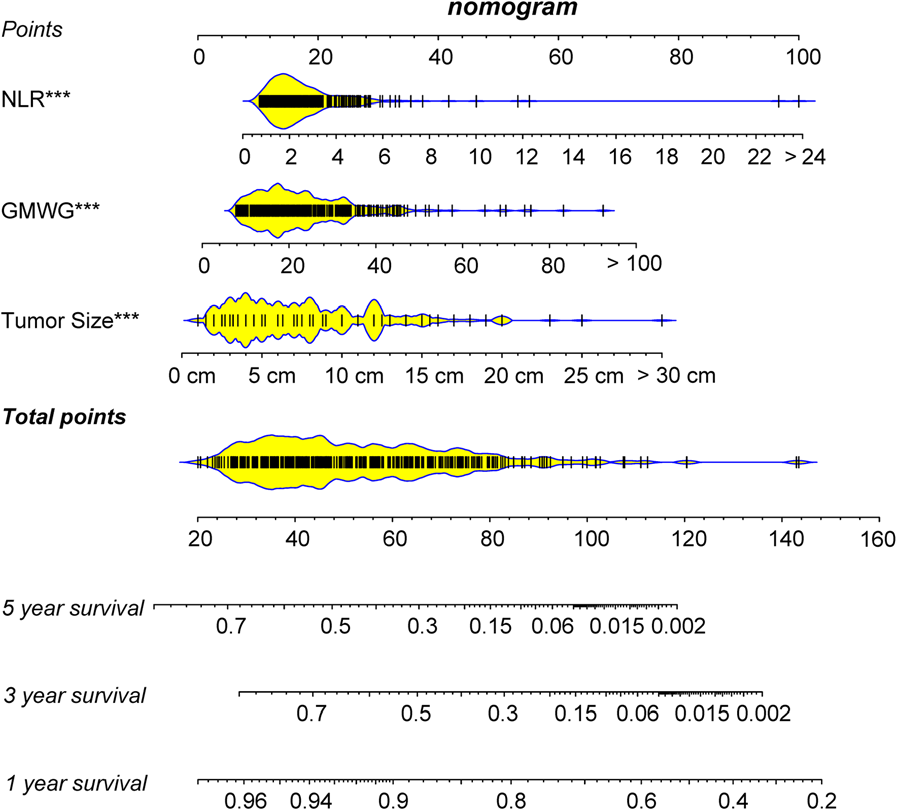
Nomogram is built to predict the overall survival. The total score is obtained according to the value of each indicator, and the survival rate corresponding to the total score is the predicted rate by nomogram

**Fig. 3:**
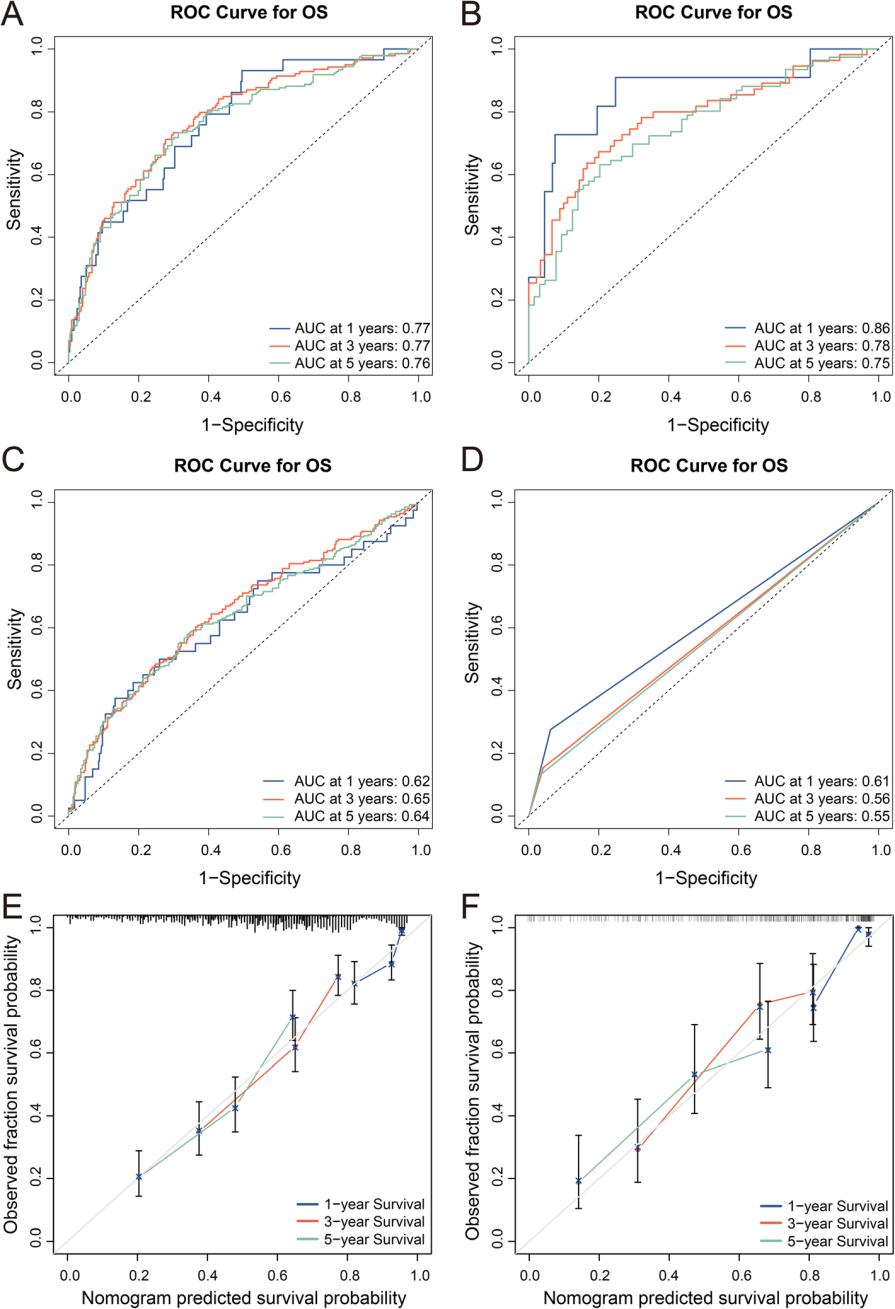
ROC curves are plotted based on different models. **A** ROC curve and AUC of the nomogram to predict 1-, 3- and 5-year overall survival in the training cohort. **B** ROC curve and AUC of nomogram in the validation cohort. **C** ROC curve and AUC of SII in the total cohort. **D** ROC curve and AUC of TNM staging system in the total cohort. Calibration curves and Kaplan–Meier curves of the nomogram. **E** Calibration curves of nomogram in the training and validation cohort **F** predict 1-, 3- and 5-year overall survival

We developed a predictive nomogram containing NLR, GMWG and tumor size, which demonstrated to be statistically significant in multivariate analysis (Fig. 2). AUC of 1-,3- and 5- year OS were 0.77 (95% CI = 0.68–0.85), 0.77 (95% CI = 0.72–0.82) and 0.76 (95% CI = 0.70–0.80) respectively in training cohort while 0.86 (95% CI = 0.73–0.99), 0.78 (95% CI = 0.70–0.86) (Fig. 3 A) and 0.75 (95% CI = 0.67–0.83) in validation cohort (Fig. 3 B). And the nomogram showed higher accuracy than any single factor (Fig. S1). The C-index of this model in training cohort was 0.70 and 0.71 in validation cohort. The results above showed nomogram demonstrate good accuracy in the prediction of overall survival.

To further verify the performance of the nomogram, we constructed a predictive model based on SII or TNM staging system in the total cohort. AUC and C-index were calculated to assess the predictive ability of different models. The 1-,3- and 5- year OS AUC of SII were 0.62 (95% CI = 0.52–0.72), 0.65 (95% CI = 0.60–0.70) and 0.64 (95% CI = 0.59–0.68) (Fig. 3 C) while the C-index was 0.59. The 1-,3- and 5- year OS AUC of TNM staging system were 0.61 (95% CI = 0.54–0.68), 0.56 (95% CI = 0.53–0.58) and 0.55 (95% CI = 0.53–0.57) (Fig. 3 D) while the C-index was 0.59. Neither of the above two models performed as well as the nomogram, and the comparison between different models showed that the nomogram had good prediction ability. Similarly, the calibration curves after 1000 times of bootstraps illustrated good agreement of predicted and observed survival outcomes (Fig. 3 E-F).

### Survival predictive ability and clinical benefit of the nomogram

**Fig. 4:**
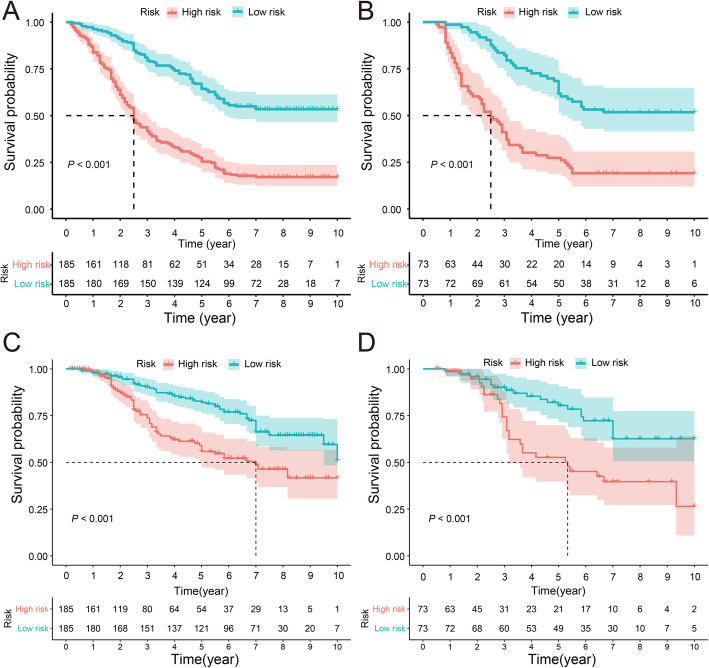
**A** Kaplan–Meier curve of overall survival in the training cohort and validation cohort **B**, Kaplan–Meier curve of disease-free survival in the training cohort **C** and validation cohort **D**, low and high risk group are divided based on the median total points

**Fig. 5:**
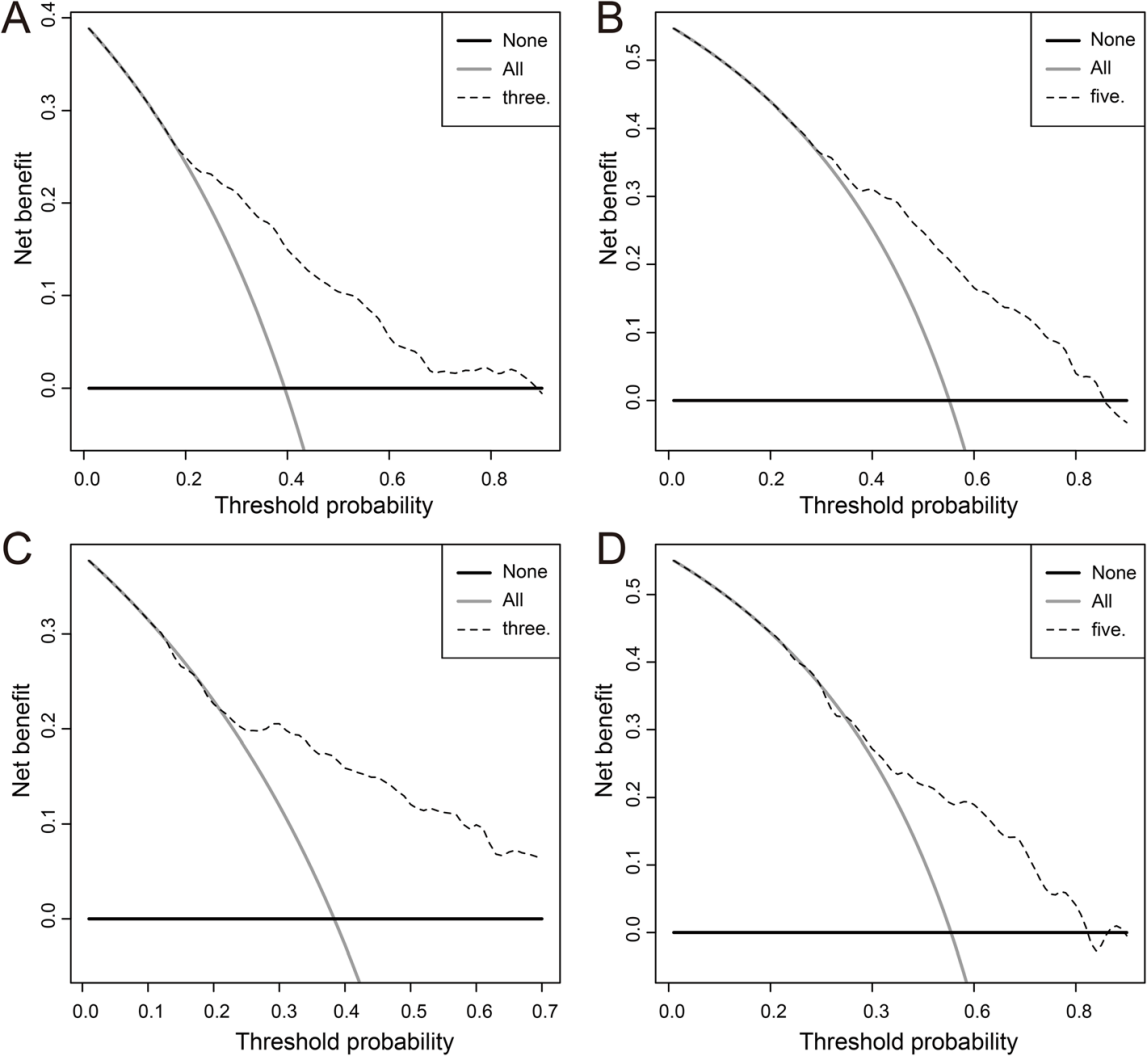
Decision curve analyses of the nomogram. **A** Decision curve of the nomogram to predict 3-year and 5-year **B** clinical net benefit in the training cohort. Decision curve of the nomogram to predict 3-year (**C**) and 5-year **D** clinical net benefit in the validation cohort. The intersection of the solid line and the dotted line with the X-axis represents the range of patients who benefit

To further explore the predictive ability of the nomogram, the total point of each patient was determined based on the monogram in both training and validation cohorts. The median point was 46.8 and 44.9 in the training and validation cohort, respectively. Then, we divided the patients into low and high risk group according to the median points and performed survival analysis via the Kaplan-Meier method. The mean overall survival times of the training cohort were 7.04 (95% CI = 6.55–7.53) years and 3.80 (95% CI = 3.34–4.26) years in the low and high risk group (*P <* 0.001) (Fig. 4 A), and in the validation cohort, the mean overall survival times of the low and high risk group were 6.99 (95% CI = 6.45–7.52) years and 3.78 (95% CI = 3.25–4.31) years respectively (*P <* 0.001) (Fig. 4 B). In the training cohort, the mean disease-free survival times of low and high risk groups were 7.99 (95% CI = 7.27–8.71) and 5.98 (95% CI = 4.99–6.96) (Fig. 4 C). In the validation cohort, the mean disease-free survival times of low and high risk groups were 8.07 (95% CI = 7.61–8.53) and 6.43 (95% CI = 5.78–7.08) (Fig. 4 D). And the survival curves based on the optimal cut-off value showed similar result, as well (Fig. S2). The results above illustrated that the nomogram has a good distinguishing ability and generalization ability. DCA curves analysis for the model shown nomogram had a higher overall net benefit across about 60% of the range of risk threshold in training cohort (Fig. 5 A-B). In the validation cohort, the DCA curve was less satisfactory but still covered about 40% of the range of risk threshold and kept a similar trend with which in training cohort (Fig. 5 C-D). In addition, absolute NRI values were determined after 1000 times of bootstraps, and we found that absolute NRI values of nomogram with GMWG included or not were all greater than 0.05 (1-year OS: NRI = 0.072; 95% CI = − 0.068–0.258, 3-year OS: NRI = 0.144 95% CI = − 0.020–0.351, 3-year OS: NRI = 0.268 95% CI = 0.024–0.474) (Table 3), which indicated nomogram included GMWG show better discrimination power than that without GMWG.

**Table 3:** Comparison of the predictive ability of nomogram include or exclude GMWG

Overall Survival	NRI (95% CI)
1 year	0.07 (−0.07–0.25)
3 year	0.14 (−0.02–0.34)
3 year	0.27 (0.02–0.46)

Note: Nomogram exclude GMWG as a reference and comparison between nomogram include GMWG and exclude GMWG

Abbreviations: *NRI* net reclassification improvement, *CI* confidence interval

## Discussion

In order to accurately predict the prognosis of patients with HCC who are undergoing radical resection, some integrated indexes have been developed, such as neutrophil to lymphocyte ratio (NLR), systemic immune-inflammation index (SII) and so on [17–19]. In this study, a new GMWG based nomogram was developed to evaluate the prognosis of HCC patients who went radical resection. The results show that its predictive ability is more optimal than that of the indexes mentioned above and TNM staging system. Among them, GMWG and NLR in the nomogram are based on the results of laboratory examination, which are convenient to access while tumor size reflects the malignant degree of the tumor from the point of pathological. We integrated the three indicators above to construct a nomogram that could predict the prognosis of postoperative patients with HCC. We assume that the routine laboratory serological examination combined with pathological characteristics could more truly reflect the tumor immune response status and tumor heterogeneity, so it has the potential to be used as an indicator of postoperative prognosis in patients with HCC.

According to the traditional view, primary liver cancer is an immunosuppressive tumor [20, 21]. Studies have shown that tumor-specific cytotoxic T cells in primary liver cancer are accompanied by high expression of inhibitory immune checkpoints such as CTLA4, PD-L1 and TIM3 [22–24], which makes T cells unable to effectively recognize tumor cells and lead to immune escape [25]. Tumor-infiltrating lymphocytes can reflect the immune response status of patients [26], however, the level of lymphocytes in peripheral blood plays a role in this as well [27]. Peripheral blood is easy to obtain, with low cost and can be continuously monitored. Hence, many studies have been carried out around the level of immune cells in patients’ peripheral blood before the operation. However, the evaluation of immune status alone can’t fully represent the anti-tumor status of patients, and the residual of normal liver cell function is also closely related to the prognosis of patients [28]. Many indicators are regarded as factors reflecting liver function in the clinic, such as AST, alanine aminotransferase (ALT) and GGT. AST and ALT are mainly distributed in normal hepatocytes [29]. When hepatocytes are damaged for various reasons, they are released into the bloodstream and lead to an increase. Although the sensitivity of AST and ALT is very high, the specificity of HCC related hepatocytes damage is not high since the inflammation and infection may also cause the increase of transaminase. Therefore, for patients with HCC, more specific indicators are needed to predict the prognosis of patients. GGT is a membrane-binding enzyme, which has long been regarded as the signal of liver cell death, especially in HCC. Previous studies have indicated that raised expression of hepatic GGT may be closely associated with the development of HCC and also suggested a poor outcome [30, 31]. Therefore, we combined WBC and GGT to build a novel indicator GMWG, which might represent immune response and residual liver function. Similar indicators have been developed in the previous study, such as granulocyte to lymphocyte ratio (GLR) and GGT to platelet ratio (GPR) [32, 33]. Rooney et al. reported an indicator which is based on the geometric mean of two markers [34]. Similarly, since these two indicators (GGT and WBC) in this study are nonnegative numbers and don’t follow a normal distribution, and we use the geometric mean to define the new indicator called GMWG, which showed predictive power for the prognosis in patients with HCC. Recently, more and more studies have illustrated the effect of tumor heterogeneity in predicting prognosis. The degree of cell differentiation and TNM staging system could influence the prognosis of HCC patients [35]. Nevertheless, both of them are determined by postoperative pathological examination. Moreover, there are some other characteristics of tumor have been proved to be closely relevant to the outcome of patients [36]. In Asin and many other areas, viral hepatitis are still the main risk factors of HCC and many patients inevitably developed to liver cirrhosis before HCC, which causes a high proportion of liver cirrhosis and leading liver cirrhosis might be a prognostic factor in the cohorts of those areas. Tumor size and tumor count are major prognostic factors in many cancers, including HCC, we could make a more accurate prediction if these indicators were taken into account. Herein, characteristics of the tumor are included to build the model. Nomogram is a convenient and readable visual tool that is widely used in the diagnosis and prognosis. The total point of the nomogram reflexes the probabilities according to the scale on a ruler. Traditional nomogram displays variables as categorical variables, which lead to the close scores of each patient. In this study, variables are analyzed and displayed as parametric variables if they already were. A more accurate score was determined and achieve a better classification, NLR, GMWG and tumor size were eventually included in the nomogram. The subsequent evaluation also shows that the nomogram has good prediction ability and generalization ability compared with other models such as SII or TNM staging system. Whereas SII, in fact, showed more ability in the prediction of recurrence rate, and the nomogram in this study is aimed to predict overall survival, the results were as expected.

There are a few limitations in this study. Most of the HCC patients in China are HBV-related, and the frequency of positive is 82.3% in this study, which differs widely from the patients in the United States, Europe, and other countries or regions [37]. Therefore, the nomogram needs to be validated in these areas. Moreover, to avoid bias, we also need to conduct prospective trials to confirm our results.

## Conclusion

In conclusion, GMWG showed the potential to be used as an indicator in the prediction of prognosis in patients with HCC. Moreover, the nomogram containing GMWG built in this study illustrated satisfaction prediction ability compared with conventional models, which may serve as a potential tool to the prediction of patients with HCC who underwent radical resection.

## Supplementary Information

**Additional file 1.** Table S1. The other clinicopathologic characteristics of patients in the training and validation cohorts. Fig. S1. ROC curves showed nomogram had more effectiveness compared with a single factor. ROC curves and AUCs of NLR, tumor size, GMWG and nomogram to predict overall survival in the training cohort (A) and validation cohort (B). Fig. S2. Overall survival and disease-free survival curves based on the best cut-off values. The optimal cut-off value (A and C) and Kaplan–Meier curve (B and D) for overall survival in the training cohort (A and B) and validation cohort (C and D). The optimal cut-off value (E and G) and Kaplan–Meier curve (F and H) for disease-free survival in the training cohort (E and F) and validation cohort (G and H).

## Data Availability

The datasets used and analyzed in the current study are available from the corresponding author upon reasonable request.

## Abbreviations

AFP: Alpha fetoprotein
ALT: Alanine aminotransferase
AST: Aspartate aminotransferase
AUC: Area under the ROC curve
CI: Confidence interval
DBIL: Direct bilirubin
DCA: Decision curve analysis
DFS: Disease-free survival
GLR: Gamma-glutamyl transpeptidase to lymphocyte count ratio
GMWG: Geometric Mean of gamma-glutamyltranspeptidase and white blood cell
HBsAg: Hepatitis B surface antigen
HCC: Hepatocellular carcinoma
LYMPH: Lymphocyte count
MVI: Microvascular invasion
NEUT: Neutrophil count
NLR: Neutrophils to lymphocytes ratio
NRI: Net reclassification improvement
OS: Overall survival
TBIL: Total bilirubin
ROC: Receiver operating characteristic
SII: systemic immune-inflammation index
WBC: White blood cell

## References

[1] Bray F, Ferlay J, Soerjomataram I, Siegel RL, Torre LA, Jemal A (2018). Global cancer statistics 2018: GLOBOCAN estimates of incidence and mortality worldwide for 36 cancers in 185 countries. CA Cancer J Clin.

[2] European Association for the Study of the Liver (2018). EASL clinical practice guidelines: management of hepatocellular carcinoma. J Hepatol.

[3] Glantzounis GK, Paliouras A, Stylianidi MC, Milionis H, Tzimas P, Roukos D, Pentheroudakis G, Felekouras E (2018). The role of liver resection in the management of intermediate and advanced stage hepatocellular carcinoma. A systematic review. Eur J Surg Oncol.

[4] Zhong JH, Ke Y, Gong WF, Xiang BD, Ma L, Ye XP, Peng T, Xie GS, Li LQ (2014). Hepatic resection associated with good survival for selected patients with intermediate and advanced-stage hepatocellular carcinoma. Ann Surg.

[5] Poon RT, Fan ST, Lo CM (2002). Long-term survival and pattern of recurrence after resection of small hepatocellular carcinoma in patients with preserved liver function: implications for a strategy of salvage transplantation. Ann Surg.

[6] Llovet JM, De Baere T, Kulik L (2021). Locoregional therapies in the era of molecular and immune treatments for hepatocellular carcinoma. Nat Rev Gastroenterol Hepatol.

[7] El-Khoueiry AB, Sangro B, Yau T (2017). Nivolumab in patients with advanced hepatocellular carcinoma (CheckMate 040): an open-label, non-comparative, phase 1/2 dose escalation and expansion trial. Lancet..

[8] Kambhampati S, Bauer KE, Bracci PM, Keenan BP, Behr SC, Gordan JD, Kelley RK (2019). Nivolumab in patients with advanced hepatocellular carcinoma and child-Pugh class B cirrhosis: safety and clinical outcomes in a retrospective case series. Cancer..

[9] Wang Y, Li J, Xia Y, Gong R, Wang K, Yan Z, Wan X, Liu G, Wu D, Shi L, Lau W, Wu M, Shen F (2013). Prognostic nomogram for intrahepatic cholangiocarcinoma after partial hepatectomy. J Clin Oncol.

[10] Liang W, Zhang L, Jiang G, Wang Q, Liu L, Liu D, Wang Z, Zhu Z, Deng Q, Xiong X, Shao W, Shi X, He J (2015). Development and validation of a nomogram for predicting survival in patients with resected non-small-cell lung cancer. J Clin Oncol.

[11] Wang T, Zhang KH (2020). New blood biomarkers for the diagnosis of AFP-negative hepatocellular carcinoma. Front Oncol.

[12] Li S, Xu W, Liao M, Zhou Y, Weng J, Ren L, Yu J, Liao W, Huang Z (2021). The significance of gamma-glutamyl transpeptidase to lymphocyte count ratio in the early postoperative recurrence monitoring and prognosis prediction of AFP-negative hepatocellular carcinoma. J Hepatocell Carcinoma.

[13] Zhang H, Zhou Y, Li Y, Qin W, Zi Y, Liu Y, Qiu X, Xu H, Liao W, Huang Z (2020). Predictive value of gamma-glutamyl transpeptidase to lymphocyte count ratio in hepatocellular carcinoma patients with microvascular invasion. BMC Cancer.

[14] Liao M, Qin W, Liao Y, Yao R, Yu J, Liao W (2019). Prognostic value of gamma-glutamyl transpeptidase to lymphocyte count ratio in patients with single tumor size ≤ 5 cm hepatocellular carcinoma after radical resection. Front Oncol.

[15] Prieto J, Melero I, Sangro B (2015). Immunological landscape and immunotherapy of hepatocellular carcinoma. Nat Rev Gastroenterol Hepatol.

[16] Stotz M, Gerger A, Eisner F, Szkandera J, Loibner H, L Ress A, Kornprat P, A Zoughbi W, Seggewies FS, Lackner C, Stojakovic T, Samonigg H, Hoefler G, Pichler M (2013). Increased neutrophil-lymphocyte ratio is a poor prognostic factor in patients with primary operable and inoperable pancreatic cancer. Br J Cancer.

[17] Hu B, Yang XR, Xu Y, Sun YF, Sun C, Guo W, Zhang X, Wang WM, Qiu SJ, Zhou J, Fan J (2014). Systemic immune-inflammation index predicts prognosis of patients after curative resection for hepatocellular carcinoma. Clin Cancer Res.

[18] Lu LH, Wei W, Li SH, Zhang YF, Guo RP (2021). The lymphocyte-C-reactive protein ratio as the optimal inflammation-based score in patients with hepatocellular carcinoma underwent TACE. Aging (Albany NY).

[19] Wang D, Bai N, Hu X, OuYang XW, Yao L, Tao YM, Wang ZM (2019). Preoperative inflammatory markers of NLR and PLR as indicators of poor prognosis in resectable HCC. PeerJ..

[20] Gajewski TF, Schreiber H, Fu YX (2013). Innate and adaptive immune cells in the tumor microenvironment. Nat Immunol.

[21] Zhang Q, He Y, Luo N, Patel SJ, Han Y, Gao R, Modak M, Carotta S, Haslinger C, Kind D, Peet GW, Zhong G, Lu S, Zhu W, Mao Y, Xiao M, Bergmann M, Hu X, Kerkar SP, Vogt AB, Pflanz S, Liu K, Peng J, Ren X, Zhang Z (2019). Landscape and dynamics of single immune cells in hepatocellular carcinoma. Cell..

[22] Zhou G, Sprengers D, Boor PPC, Doukas M, Schutz H, Mancham S, Pedroza-Gonzalez A, Polak WG, de Jonge J, Gaspersz M, Dong H, Thielemans K, Pan Q, IJzermans JNM, Bruno MJ, Kwekkeboom J (2017). Antibodies against immune checkpoint molecules restore functions of tumor-infiltrating T cells in hepatocellular carcinomas. Gastroenterology..

[23] Darvin P, Toor SM, Sasidharan Nair V, Elkord E (2018). Immune checkpoint inhibitors: recent progress and potential biomarkers. Exp Mol Med.

[24] Ma J, Zheng B, Goswami S, Meng L, Zhang D, Cao C, Li T, Zhu F, Ma L, Zhang Z, Zhang S, Duan M, Chen Q, Gao Q, Zhang X (2019). PD1(hi) CD8(+) T cells correlate with exhausted signature and poor clinical outcome in hepatocellular carcinoma. J Immunother Cancer.

[25] Dunn GP, Bruce AT, Ikeda H, Old LJ, Schreiber RD (2002). Cancer immunoediting: from immunosurveillance to tumor escape. Nat Immunol.

[26] Zheng C, Zheng L, Yoo JK, Guo H, Zhang Y, Guo X, Kang B, Hu R, Huang JY, Zhang Q, Liu Z, Dong M, Hu X, Ouyang W, Peng J, Zhang Z (2017). Landscape of infiltrating T cells in liver Cancer revealed by single-cell sequencing. Cell..

[27] Estrade F, Lescure C, Muzellec L, Pedrono M, Palard X, Pracht M, le Sourd S, Rolland Y, Uguen T, Garin E, Edeline J (2020). Lymphocytes and neutrophil-to-lymphocyte ratio variations after selective internal radiation treatment for HCC: a retrospective cohort study. Cardiovasc Intervent Radiol.

[28] Cieslak KP, Baur O, Verheij J, Bennink RJ, van Gulik TM (2016). Liver function declines with increased age. HPB (Oxford).

[29] Trefts E, Gannon M, Wasserman DH (2017). The liver. Curr Biol.

[30] Liu Y, Zhang Q, Yang X, Li Y, Zhu B, Niu S, Huang Y, Hu Y, Wang X (2019). Effects of various interventions on the occurrence of macrovascular invasion of hepatocellular carcinoma after the baseline serum gamma-glutamyltransferase stratification. Onco Targets Ther.

[31] Shen J, Tang L, Zhang X, Peng W, Wen T, Li C, Yang J, Liu G (2019). A novel index in hepatocellular carcinoma patients after curative hepatectomy: albumin to gamma-glutamyltransferase ratio (AGR). Front Oncol.

[32] Li J, Liao Y, Suo L, Zhu P, Chen X, Dang W, Liao M, Qin L, Liao W (2017). A novel prognostic index-neutrophil times gamma-glutamyl transpeptidase to lymphocyte ratio (NγLR) predicts outcome for patients with hepatocellular carcinoma. Sci Rep.

[33] Dai T, Deng M, Ye L, Liu R, Lin G, Chen X, Li H, Liu W, Yang Y, Chen G, Wang G (2020). Prognostic value of combined preoperative gamma-glutamyl transpeptidase to platelet ratio and fibrinogen in patients with HBV-related hepatocellular carcinoma after hepatectomy. Am J Transl Res.

[34] Rooney MS, Shukla SA, Wu CJ, Getz G, Hacohen N (2015). Molecular and genetic properties of tumors associated with local immune cytolytic activity. Cell..

[35] Bruix J, Reig M, Sherman M (2016). Evidence-based diagnosis, staging, and treatment of patients with hepatocellular carcinoma. Gastroenterology..

[36] Cong WM, Bu H, Chen J, Dong H, Zhu YY, Feng LH, Chen J, Committee G (2016). Practice guidelines for the pathological diagnosis of primary liver cancer: 2015 update. World J Gastroenterol.

[37] Chen Y, Tian Z (2019). HBV-induced immune imbalance in the development of HCC. Front Immunol.

