# Supplementary material for: Predictive potential of Nomogram based on GMWG for patients with hepatocellular carcinoma after radical resection
Source: BMC Cancer. 2021 Jul 15;21:817. doi: 10.1186/s12885-021-08565-2 (PMC8283989; doi:10.1186/s12885-021-08565-2)
Supplement: Supplementary file 1 — Additional file 1. Table S1. The other clinicopathologic characteristics of patients in the training and validation cohorts. Fig. S1. ROC curves showed nomogram had more effectiveness compared with a single factor. ROC curves and AUCs of NLR, tumor size, GMWG and nomogram to predict overall survival in the training cohort (A) and validation cohort (B). Fig. S2. Overall survival and disease-free survival curves based on the best cut-off values. The optimal cut-off value (A and C) and Kaplan–Meier curve (B and D) for overall survival in the training cohort (A and B) and validation cohort (C and D). The optimal cut-off value (E and G) and Kaplan–Meier curve (F and H) for disease-free survival in the training cohort (E and F) and validation cohort (G and H). [file 12885_2021_8565_MOESM1_ESM.doc]

**Table S1. The other clinicopathologic characteristics of patients in the training and validation cohorts**

| **Parameter** | **Training cohort** |  | **Validation cohort** | ***p* value** |
| --- | --- | --- | --- | --- |
|  | **(n = 370)** |  | **(n = 146)** |  |
| HBsAb | 20.06±78.57 |  | 6.64±37.18 | 0.049* |
| HBeAg | 1.76±12.83 |  | 1.30±8.22 | 0.685 |
| HBeAb | 8.84±19.17 |  | 6.87±12.37 | 0.252 |
| HBcAb | 18.71±53.18 |  | 12.73±28.82 | 0.199 |
| WBC (×10^9^/L) | 6.35±2.25 |  | 6.53±2.67 | 0.108 |
| Albumin (g/L) | 38.54±5.03 |  | 38.85±4.33 | 0.046* |
| [Globulin](javascript:void(0);) (g/L) | 31.36±5.71 |  | 30.75±5.69 | 0.192 |
| TBIL (μmol/L) | 16.05±25.31 |  | 16.31±21.32 | 0.622 |
| DBIL (μmol/L) | 7.35±19.38 |  | 7.04±18.80 | 0.783 |
| ALT (U/L) | 44.24±41.72 |  | 47.48±50.48 | 0.037* |
| AST (U/L) | 45.56±42.83 |  | 46.76±55.14 | 0.005* |
| Ishak score: 0/1/2/3 (n) | 34/130/135/71 |  | 11/62/44/29 | 0.379 |

**Note:** **p-*value indicates statistically significant.

**Abbreviations:** n, number of patients; HbsAb, antibody to hepatitis surface antigen; HBeAg, hepatitis B e-antigen; HBeAb, antibody to hepatitis B e-antigen; HBcAb, antibody to hepatitis B core antigen; WBC, white blood cell; TBIL, total bilirubin; DBIL, [direct](javascript:void(0);) [bilirubin](javascript:void(0);); ALT, alanine aminotransferase; AST, aspartate aminotransferase;

**Figure S1.** Predictive ability of the nomogram and a single factor.

**
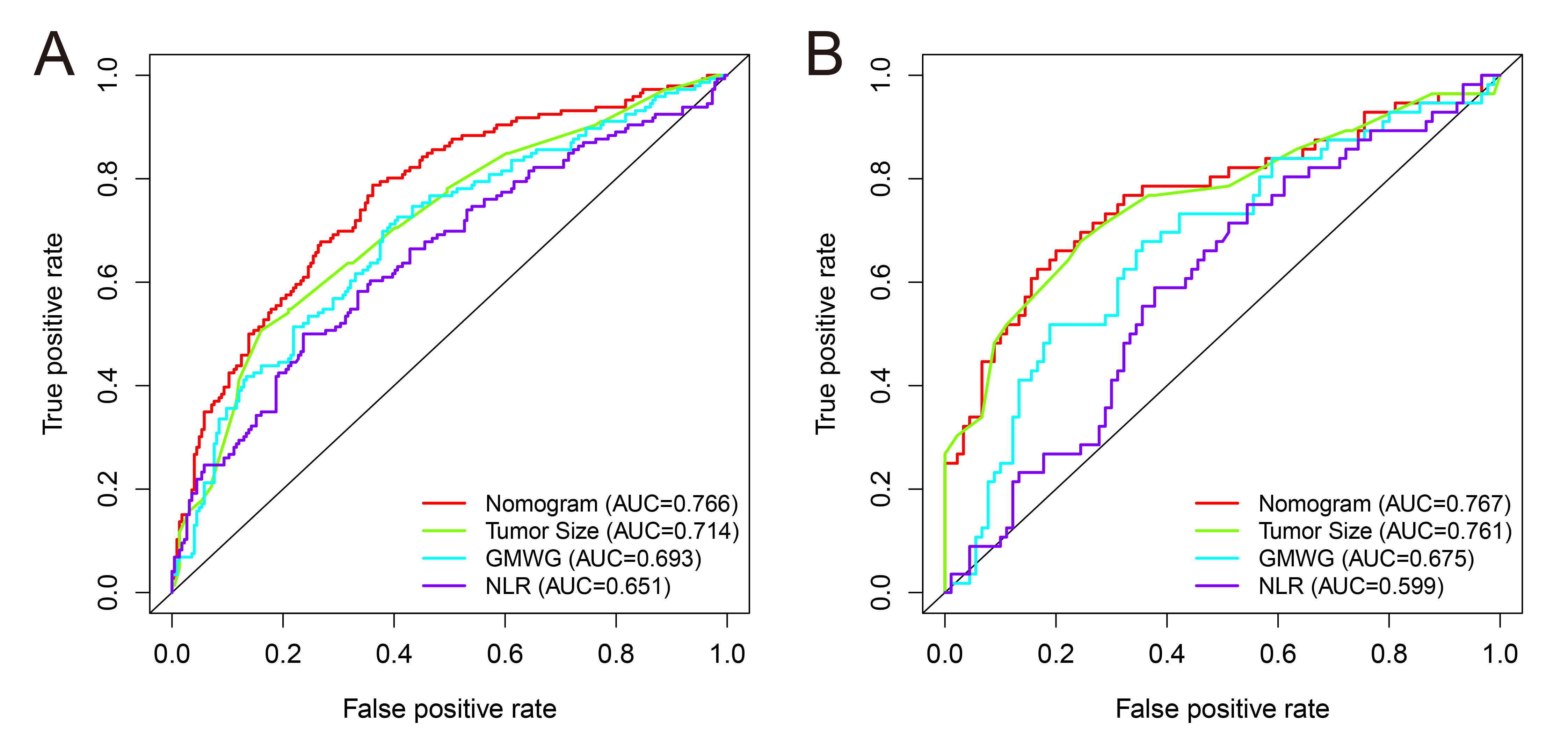
**

Figure S1: ROC curves showed nomogram had more effectiveness compared with a single factor. ROC curves and AUCs of NLR, tumor size, GMWG and nomogram to predict overall survival in the training cohort (A) and validation cohort (B).

**Figure S2.** Nomogram predicted survival based on the optimal cut-off values.

**
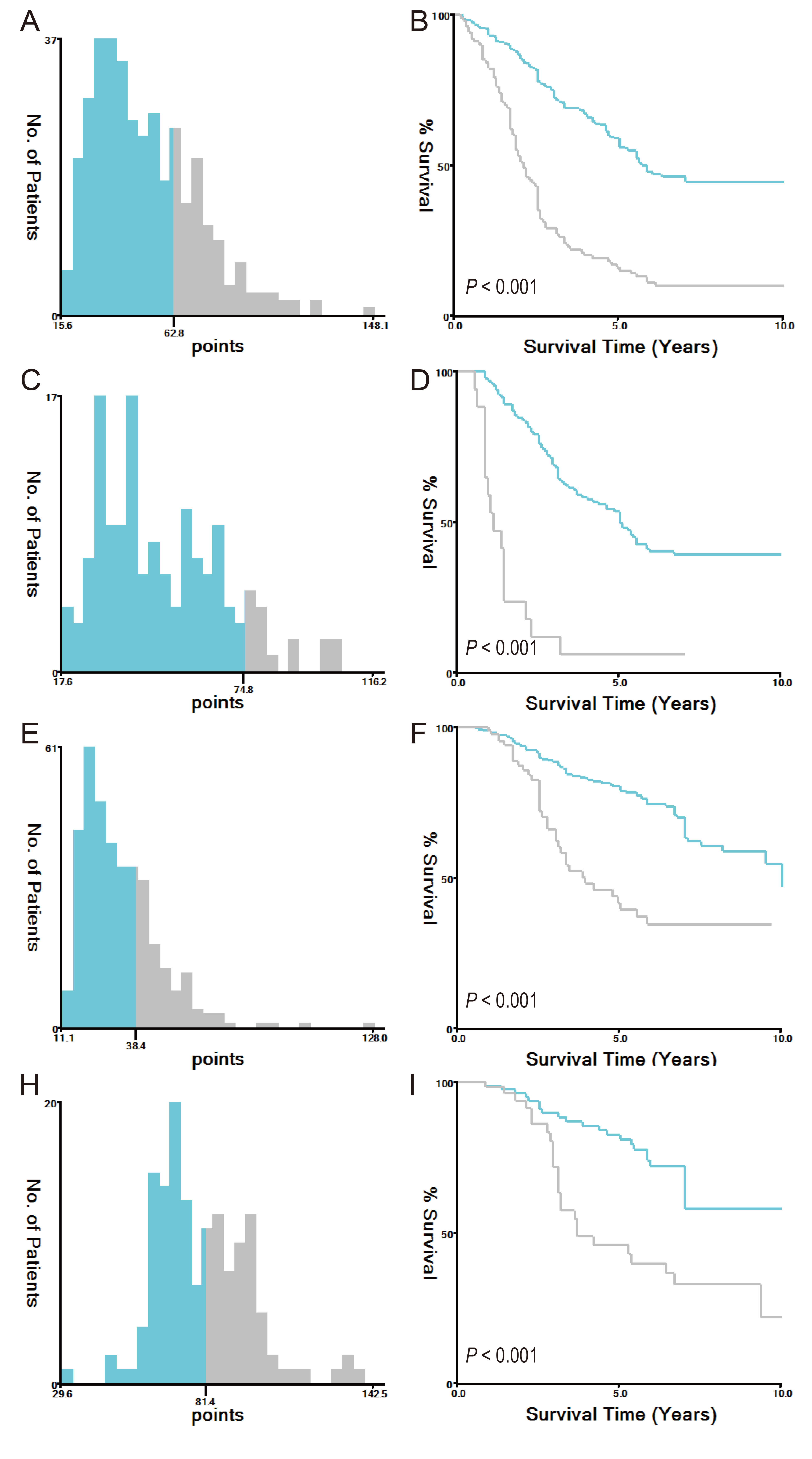
**

Figure S2: Overall survival and disease-free survival curves based on the best cut-off values. The optimal cut-off value (A and C) and Kaplan–Meier curve (B and D) for overall survival in the training cohort (A and B) and validation cohort (C and D). The optimal cut-off value (E and G) and Kaplan–Meier curve (F and H) for disease-free survival in the training cohort (E and F) and validation cohort (G and H).
